# Public Priorities in Women's Health: Analysis of Request for Information Published to Inform “Advancing NIH Research on the Health of Women: A 2021 Conference”

**DOI:** 10.1089/jwh.2022.0488

**Published:** 2023-06-06

**Authors:** Elizabeth Barr, Samia Noursi, Erik Roodzant, Amelia Ubesie, Shilpa Amin, Nikeya Macioce, Damiya Whitaker, Janine A. Clayton, Sarah M. Temkin

**Affiliations:** ^1^ National Institutes of Health, Office of Research on Women's Health, Bethesda, Maryland, USA.; ^2^ Booz Allen Hamilton, McLean, Virginia, USA.; ^3^ Scientific Consulting Group, Gaithersburg, Maryland, USA.

**Keywords:** women's health, patient perspective, maternal morbidity and mortality, chronic conditions, cervical cancer

## Abstract

**Objectives:**

To assist with planning a congressionally requested conference on women's health research, the National Institutes of Health (NIH) Office of Research on Women's Health (ORWH) invited comments to characterize public concerns related to any or all of the specified public health issues: maternal morbidity and mortality (MMM); stagnant rates of cervical cancer survival; and the growing incidence of chronic debilitating conditions in women (CDCW). This analysis summarizes public priorities in women's health research.

**Materials and Methods:**

All comments received in response to a request for information were open coded and a master list of keywords was created, and comments were categorized. Comments addressing CDCW were categorized using a conceptual framework developed by the NIH.

**Results:**

Two hundred forty-seven comments were coded and analyzed. One hundred four comments (42%) addressed MMM; 182 comments (73%) discussed CDCW; and 27 comments (10%) addressed cervical cancer. Comments focused on CDCW most frequently addressed female-specific conditions (83%). The 10 most frequently identified keywords in order of frequency from the manual coding were as follows: (1) MMM, (2) racial disparities, (3) access to care, (4) provider training, (5) mental health, (6) Black or African American women, (7) screening, (8) quality of care, (9) time to diagnosis, and (10) social determinants of health.

**Conclusions:**

Comments demonstrate a broad range of concerns related to the health of women, including MMM, CDCW, and cervical cancer. A wide array of commenters included patients, advocacy groups, and academic and professional organizations originating from geographically diverse locations. These comments reflect a strong desire from the public to prioritize research on the health of women.

## Introduction

The National Institutes of Health (NIH) Revitalization Act of 1993 created the Office of Research on Women's Health (ORWH) in response to concerns about the lack of inclusion of appropriate numbers of women in clinical research.^1^ Today, in terms of overall enrollment to NIH-supported clinical trials, women are enrolled at similar rates to men and collaborative efforts have resulted in significant advances in research focused on the health of women.^2^ Yet, several conditions that have high morbidity for women, including autoimmune disorders and female-specific conditions, remain underrepresented in funding level within the NIH research portfolio.^3^

In their fiscal year (FY) 2021 reports, the House^4^ and Senate^5^ Appropriations Committees requested that the NIH convene a conference to evaluate research currently underway related to the health of women, specifically regarding the following three topics: rising rates of maternal morbidity and mortality (MMM); rising rates of chronic debilitating conditions in women (CDCW); and stagnant cervical cancer survival rates.^6^

These three focused topics designated by Congress represent significant public health concerns in the United States. In 2020, the overall maternal mortality rate in the United States was 23.8 deaths per 100,000 live births, considerably higher than maternal mortality rates in peer countries.^7,8^ Women in the United States more commonly have a diagnosis of a chronic condition, as well as multimorbidity—the simultaneous occurrence of two or more diseases that may or may not share a causal link—compared to men.^9^ Despite the widespread availability of effective cervical cancer screening and prevention with the Food and Drug Administration's approved human papillomavirus (HPV) vaccine, the age-adjusted death rate from cervical cancer decreased by only 0.7% annually between 2009 and 2018^10^ and has even increased recently for those women living in the lowest-income counties.^11^ Significant racial disparities in each of these conditions exist.^7,12,13^

In response to the congressional request for public input to inform the conference, on July 1, 2021, the ORWH published a Request for Information (RFI) in the *Federal Register* (86 FR 35099).^14^ The RFI invited comments from the extramural scientific community, professional societies, and the general public to assist with identifying research gaps and pitfalls in clinical practices and to obtain real-life testimonial experiences (direct or indirect) related to any or all of the specified public health issues. The objective of this analysis is to summarize the public comments.

## Materials and Methods

On July 1, 2021, the ORWH published a RFI in the *Federal Register* (86 FR 35099).^14^ Comments were invited electronically to a dedicated email inbox, WHCC@nih.gov The comment submission period remained open until September 15, 2021.

Duplicate comments were removed, and the full set of unique comments was compiled and open coded. An initial list of keywords was iteratively created by four reviewers, and the keyword list was further refined into a master list of 150 keywords by two reviewers. Each comment then was independently coded by two reviewers using the keyword master list, commenter type, and RFI topic.

Comments addressing CDCW were further categorized using the conceptual framework developed by the NIH in conference preparation (1) as female specific; (2) more common in women or morbidity is higher in women; (3) occur in both sexes, potentially understudied in women; or (4) high morbidity for women.^6^ All comments also were reviewed and coded for clinically relevant elements: screening, prevention, treatment, basic research, implementation, and disparities. The coding team consolidated individual reviews. When discrepancies existed between two reviewers' coding, a subset of the coding team discussed and determined final codes by consensus. ORWH staff described themes and trends in the public comments and synthesized the recommendations provided by commenters.

An additional quantitative analysis of comment text was performed using ProSuite,^15^ a content analysis software. ProSuite counted the number of times words and phrases appeared in the comments. The word frequencies then were sorted to highlight terms and phrases used most often, excluding common English words (*e.g.*, “and,” “but,” “or”). This analysis was used to verify the choice of keywords.

## Results

A total of 260 comments were received by ORWH *via* electronic mail. After excluding 13 duplicate comments, 247 comments were included in the analysis. Comments were received from 56 individuals identifying as researchers, 49 members of the public, 40 individuals writing to provide patient testimony, 36 advocacy groups, 34 health care providers, 13 professional societies, 10 industry representatives, and 8 government agency officials. The location of 111 comments was not disclosed; however, the remaining 136 responses were received from individuals located in 36 U.S. states, Sweden, and Switzerland. The largest number of comments were received from California (*n* = 22), followed by New York (*n* = 16) and Maryland (*n* = 15).

**FIG. 1.**
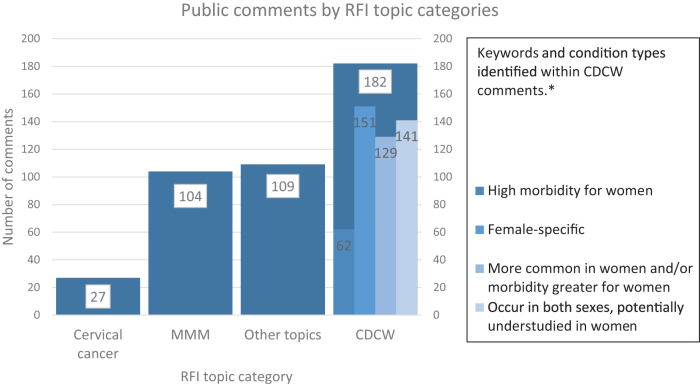
RFI topics and other emergent themes. *Total KW #s do not align to total # of comments as comments could address multiple KWs. KW, keyword; CDCW, chronic debilitating conditions in women; MMM, maternal morbidity and mortality; RFI, request for information.

Most comments focused on at least one of the topics specified in the RFI: 73% (*n* = 182) discussed CDCW, 42% (*n* = 104) addressed MMM, and 10% (*n* = 27) addressed cervical cancer (Fig. 1). Selected comments on each topic are provided in Table 1. In addition, women's health topics not specified in the RFI were raised in 44% (*n* = 109) of comments. A wide range of clinically relevant elements were identified through manual coding, with commenters addressing basic research (*n* = 123), treatment (*n* = 121), screening (*n* = 113), disparities (*n* = 98), prevention (*n* = 86), and implementation (*n* = 51).

**Table 1.** Selected Comments from Responses to the Request for Information (86 FR 35099) to Inform Advancing National Institutes of Health Research on the Health of Women: A 2021 Conference

Commenter category	Comment
Women's health research	
Advocacy group	*Methodology for properly defining women's health research … will enable NIH to accurately categorize its research and stakeholders to work more effectively with ORWH and NIH Institutes and Centers to fill existing research gaps and advance women's health.* *Including sex as a biological variable is an important step in redressing this funding gap but does not address the deficit of funded research specific to the unique biological, psychosocial, and social-political health experiences of women and girls, who constitute half the U.S. population. We urge NIH to radically expand funding on women's health.* *The NIH should identify and provide new opportunities for trainees and young investigators to engage in research related to sex- and gender-specific conditions. In addition to providing targeted funded resources for women's health research, we must also consider the human capital required to address sex- and gender-based health disparities. In order to create a sustainable pipeline for women's health research, dedicated resources and training mechanisms should be developed to engage the next generation of leaders in science and medicine who are cognizant of both the biological and social determinants of health imparted by sex and gender. By cultivating a robust workforce which can address sex- and gender-based research questions, we will be able to minimize gaps in research practice and health care and generate new discoveries and real-world innovation.* *Dedicated resources and training mechanisms should be developed to engage the next generation of leaders in science and medicine who are cognizant of both the biological and social determinants of health imparted by sex and gender. By cultivating a robust workforce which can address sex- and gender-based research questions, we will be able to minimize gaps in research practice and health care and generate new discoveries and real-world innovation.*
Researcher	*I am an epidemiology PhD student at [a midwestern university] researching endometriosis. I am also an endometriosis patient. I chose this research because I was met with constant roadblocks, poor information, and stonewalling while trying to get my own diagnosis which took 15years of consistent requests for help, at least 20 doctors, and invasive testing before finally getting surgery and a diagnosis. My story is not unusual. I felt unheard and like this topic has received little attention from researchers. I want to change that, and I hope the NIH will also recognize that this disease affects so many people who go into clinician offices everyday just to be told that the symptoms are all in our head if birth control pills don't work.* *There has not been systematic investment on the part of the NIH and ORWH into examining the effects of gender across biomedical research topics. In failing to properly address gender as a fundamental health influence, NIH is missing ripe, low-hanging fruit for remedying women's health disparities… [The NIH should] develop a gender-focused NIH-wide policy equivalent to SABV (i.e., Gender as a Sociocultural Variable, GASV). Current research does not adequately explore the ways in which gender norms, traits, and roles impact health, either with regard to individuals' risks and outcomes or to their interaction with the health care system.* *To increase the portfolio of research on social and structural determinants of health with an eye towards achieving gender and racial equity…, there is a need to increase support for critical stakeholders in the process, including[…]Increasing the racial and gender diversity of principal investigators and study section members [and] Adding meaningful patient engagement and/or community engagement as scorable criteria in the grant review process. By systematically requiring researchers to consider patient perspectives and/or build connections to community partners, NIH can increase the potential for its funded projects to have timely and relevant impact on communities.*
Nonprofit organization	*Including sex as a biological variable is an important step in redressing this funding gap but does not address the deficit of funded research specific to the unique biological, psychosocial, and social-political health experiences of women and girls, who constitute half the U.S. population. We urge NIH to radically expand funding on women's health.*
Patient	*[M]ale doctors and female patients may not communicate as effectively as nonmale doctors (female and transgender doctors) and female patients. What gets communicated between the doctor–patient dyad might affect the care levels administered and received, respectively. For instance, male doctors may often pathologize women's symptoms as psychological in nature when they are seeking physiological care for heart attack risk or stroke risk factors and assessments. The female–female doctor–patient dyad might work better under such conditions because (a) female doctors may understand the needs of female patients better, and (b) male doctors may not have been trained to deal with their potential implicit or explicit biases against female patients.*
MMM	
Patient	*I will never forget learning about [patient] and her family and what has happened to so many other Black women and other women of color. As a Black woman … and a 26-year-old who dreams of having children someday, I fear this may also be my reality. This reality must change.*
Health care provider	*My patient population is a majority Black and deeply [affected] by racial inequities in health care and environmental health from a system of institutionalized racism. I see how this negatively [a]ffects their obstetric care every day … [I] would love to see funding that addresses the lack of health providers that are culturally congruent with the population. Many of my patients lack access to care or engagement with care due to a history of racism, but seeing a health provider that looks like them could make the difference. I would love NIH funding to research doulas and pregnancy health navigators who are from the same communities as our patients and see the differences in obstetric care and outcomes. Many other complex conditions, like cancer, have health navigators that help someone through a complex health system to make sure appointments, diagnostics, and treatments aren't missed.*
Advocacy group	*Many medical conditions disproportionately impact Black pregnant and birthing people. Addressing implicit bias in medicine—through trainings and by centering the voices of BIPOC organizations, professionals, and patients—is a critical component of improving health outcomes for BIPOC people and eliminating racial disparities in pregnancy-related outcomes. [Our organization] encourages intentional action ‘to support implicit bias training for all health care providers and support staff.*
Researcher	*Access to midwives, doulas, and freestanding birthing centers, all of which have demonstrated superior care and outcomes for Black birthing people and may be particularly adept at caring for the cultural and social-emotional needs of Black birthing people and their families, must be expanded.*
Professional society	*… specific research area recommendations for consideration [include]… trans/inter-generational trauma, adverse experiences leading to fetal/maternal programming of HPA [hypothalamic-pituitary axis], stress, [and] chronic conditions later in life*
Chronic debilitating conditions in women	
Patient	*More research is needed to inform clinical practice and improve diagnosis and treatment. Both my sisters and I were misdiagnosed for YEARS when we were suffering from endometriosis (my two sisters) and recurrent ovarian cysts (me). We were told the pain was “normal,” told it was kidney stones, told it was IBS, etc., etc. My sister was only diagnosed once she experienced ovarian torsion and underwent surgery and almost lost her right ovary. In all three of our cases, providers told us there just isn't enough evidence to understand the conditions and lead to more accurate and timely diagnoses and treatment.* *I am a DES daughter. My mother took the drug diethylstilbestrol when she was pregnant with me in 1960/1961. I have had long-lasting effects, including infertility, premature births, extremely painful menstrual periods, and the fear of what else will happen as I age.* *Since there are many doctors who don't see people with lupus regularly, clinical practice guidelines that explain the heterogeneity of lupus and provide guidance for treatment that focus[es] on the individual needs of each patient, might help others avoid what my daughter had to endure.”* *I am the mother and part-time caretaker of a recently diagnosed daughter with ME/CFS who has endured decades-long suffering in search of compassionate and evidence-based treatment for this horribly disabling and stigmatized disease.* *My rheumatologist in the 1990s told me I had Sjögren's. He told me that I would have dry mouth, dry eyes, and would probably want to use estrogen cream. I have had Parkinson's for 3years. My neurologist and arthritis doctor tell me that most of my aches and pains are from the progression of Sjögren's. All my Parkinson's contacts tell me there is very little research being done on that disease. I hope more can be done about stopping the progression and find a cure.*
Independent researcher	*There are roughly three times as many diseases whose funding pattern favors males (the disease affects mainly women and is underfunded or affects mainly men and is overfunded) as there are diseases whose funding pattern favors females; funding is measured relative to disease burden. The degree of funding bias for diseases that favor males is roughly twice as great as that for diseases that favor females. In other words, not only are there roughly three times as many diseases whose funding is biased toward males, but the degree of funding bias for those diseases is roughly twice as great.*
Professional society	*The underpinnings of the pathology of PCOS, its genetic and epigenetic origins, as well as the appropriate treatments require additional focus and investigation, as there is a significant funding gap in research fostering our understanding of the etiology of the condition and in the generation of new effective therapies.*
Advocacy group	*While there are numerous gaps in research related to sex and gender with regards to pain, we write to request that NIH prioritize research in three specific areas: disparities and inequities in the diagnosis and treatment of painful conditions in women across the age spectrum; sex-based differences in the role of immune cells in pain signaling, progression, and chronicity (in humans); and persistent pain examined from a systems biology perspective that concurrently considers neurologic, immune, and endocrine influences.* *The development of biomedical HIV prevention interventions has lagged for women compared to men, with the most egregious example being [a pharmaceutical company's] drug for [pre-exposure prophylaxis (PrEP)] … which was only approved for use in men because the company failed to conduct a study in women.* *Women are not a monolithic population, and many conditions that disparately affect women are more dominant in women of certain races: rheumatoid arthritis, for example, unequally affects women of color (especially Black and Latina women); fibroids disproportionately affect Black women; Asian women have a higher incidence of endometriosis; and Native American women are at higher risk for migraine.* *Narratives are compelling, and stories can often provide a more complete picture of a woman's health than a single data point. The Office of Research on Women's Health is uniquely positioned to consider research that is not limited to one organ system or disease state, but rather considers myriad factors that impact health and well-being and multiple diagnoses. As a patient-centered advocacy organization, [we] encourage ORWH to incorporate women's lived experiences and stories into your work, whether that be through more qualitative research studies and other unique study designs, as well as through how you communicate the results and impact of the research you lead.*
Cervical cancer survival	
Medical device company	*[We] recommend advancing self-sampling and reporting extended genotyping as policies that can begin to counter the stagnant cervical cancer survival rates … We recommend that NIH and CDC partner to advance a cervical cancer registry that will enable health care providers, researchers, and policymakers to monitor the prevalence of HPV genotypes in order to close gaps in care.*
Professional society	*Although Black women have seen a decrease in cervical cancer incidence and mortality overall, they continue to have a higher incidence than non-Hispanic Whites. Hispanic women also continue to have a higher incidence rate than [non-Hispanic] Whites. The causes of these disparities remain elusive but are likely driven by multiple factors. An important component of efforts [to increase cervical cancer screening and HPV vaccination] is increased attention to social determinants of health and their impact on cancer incidence and mortality, cervical cancer screening, and HPV vaccination practices in historically excluded and marginalized populations.*
Health care provider	*Cervical cancer research has long been underfunded by the NIH but it disproportionately affects minority women. With the disappointing results of the latest adjuvant trail and stagnant survival rates for locally advanced disease, it is clear that funding is needed to explore novel treatments such as therapeutic vaccines and immunotherapy either separately or in combination. I hope that the NIH will finally prioritize cervical cancer patients and researchers (including WOC) to improve these long stagnant outcomes.*

BIPOC, Black, Indigenous, and People of Color; DES, diethylstilbestrol; HPV, human papilloma virus; IBS, irritable bowel syndrome; ME/CFS, myalgic encephalomyelitis/chronic fatigue syndrome; MMM, maternal morbidity and mortality; NIH, National Institutes of Health; ORWH, Office of Research on Women's Health; PCOS, Polycystic Ovary Syndrome; PrEP; SABV, Sex as a Biological Variable; WOC, women of color.

**FIG. 2.**
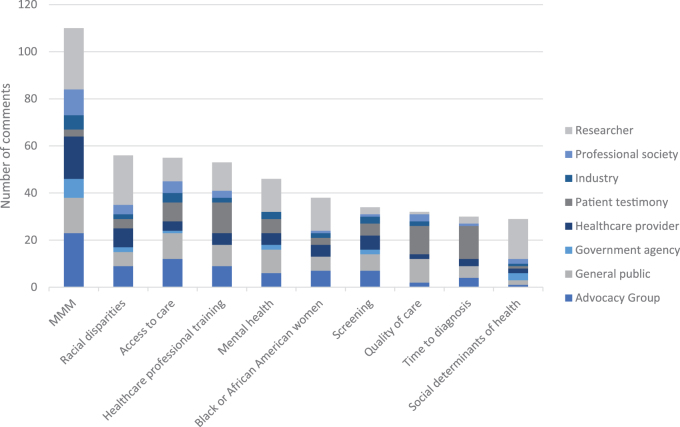
Ten most frequently identified KWs, by commenter type.

The 10 most frequently identified keywords in order of frequency from the manual coding were as follows: (1) MMM, (2) racial disparities, (3) access to care, (4) health care professional training, (5) mental health, (6) Black or African American women, (7), screening, (8) quality of care, (9) time to diagnosis, and (10) social determinants of health (Fig. 2).

**FIG. 3.**
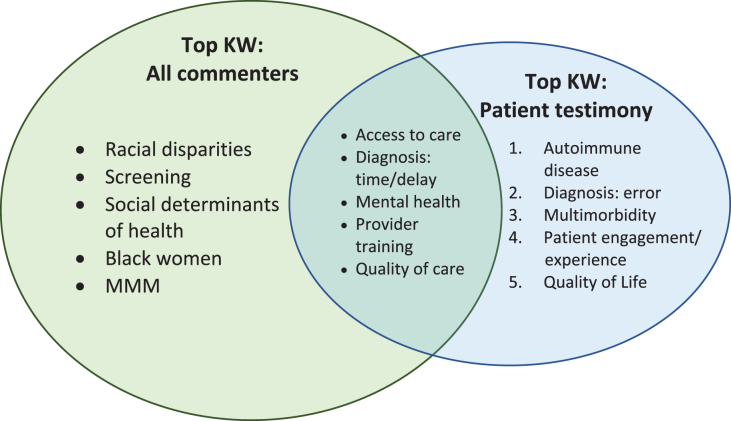
Overlap between top KWs in all comments and top KWs in patient testimony.

Patient testimonies submitted in response to the RFI added nuance and depth to the comment portfolio. Half of the keywords that appeared most frequently in patient testimonies were not well represented in comments from other groups (Fig. 3). Patient testimonies emphasized a perceived lack of interest in the health care needs of women on the part of the medical establishment. Comments from several advocacy groups echoed this sentiment. Patient testimonies frequently detailed health care experiences—many of them traumatic—with rare, understudied, and/or female-specific conditions. Several comments centered around the need for enhanced training in these conditions for health care practitioners and increased awareness of the roles of sex and gender on health and disease.

Commenters representing diverse interests urged structural changes to correct gender inequity in NIH funding patterns (*e.g.*, the underfunding of research in female-specific conditions) and in the NIH women's health research agenda (*e.g.*, increased funding for gender-based conditions, such as intimate partner violence). Comments from researchers emphasized that a long-standing lack of funding for research on gender's influences on health has inhibited scientific progress and upheld gendered health disparities. Targeted funding opportunities for women's health topics were suggested as a potential remedy.

The landmark 2016 Sex as a Biological Variable (SABV)^16^ policy was noted as an important step in understanding sex differences. Commenters recommended that the NIH develop additional policies modeled after SABV to address gender as a sociocultural and political variable. Increased NIH investment in women's health research was cited repeatedly as essential to improving the health of all women.

Commenters also raised the concept of intersectionality, or how socially determined categories—such as race and gender—overlap and interact to create disparate outcomes for individuals and communities. Structural racism, implicit bias, provider bias, and racial disparities were mentioned in RFI comments as areas of concern for women's health as well. Specifically, the needs of Black women were referenced in 24 comments and the needs of women of color in 16 comments. Racism was explicitly referenced in 18 comments as a factor preventing women from accessing care, being screened for certain diseases, and having their health concerns taken into consideration. RFI comments also advocated for improvements to care settings, specifically with respect to freedom from bias for members of the lesbian, gay, bisexual, transgender, and queer (LGBTQ+) community, and for improved data collection to better serve these populations.

### Maternal morbidity and mortality

Comments addressing MMM identified gaps in the care of pregnant women and birthing people; called for new research and programs to provide solutions to the MMM crisis; and articulated the importance of holistic, community-based care to improve entrenched racial disparities in MMM. Specifically, commenters recommended the need for new research to address the higher rates of MMM in women and pregnant people who are Black, Indigenous, and People of Color. Patient testimony reflected the urgency of the maternal mortality crisis and highlighted both the immediate and downstream impacts of this crisis.

Comments related to pregnancy and labor focused on labor induction, managing labor, and addressing complications. Eight comments cited the benefits of doulas during pregnancy, birth, and postpartum. Doulas and community-based birth support were mentioned in the context of the health of women of color, and commenters specifically identified the importance of doula care for Black women, who are at higher risk for pregnancy-related complications and death than White women. Interventions aimed at increasing cultural congruence in birth teams and access to alternate care delivery models, where evidence has demonstrated improved outcomes for Black birthing people, were advocated for by public health researchers, providers, and patients. Comments advocated for improved training to support implicit bias reduction for all health care professionals to mitigate the effects of racism on birth outcomes for Black pregnant patients.

Contraception access was cited as another element of health equity, allowing women to manage and space pregnancies and improve birth outcomes. Commenters described the value of readily available contraception, in multiple forms, so that women have access to what is easiest and best meets their individual needs and preferences. Access to high-quality reproductive health care, including abortion, was highlighted as an important option for pregnant people in the United States, for both medical and elective motivations.

### Chronic debilitating conditions in women

Seventy-three percent of comments (*n* = 182) discussed CDCW. Comments spanned the categories defined within the CDCW framework. Specific conditions mentioned in CDCW comments were identified 483 times (Table 2). These mentions were further classified as female specific (31%); more common in women and/or morbidity is higher in women (27%); potentially understudied in women (27%); and high morbidity for women (13%).

**Table 2.** Keyword Frequency^a^ Within Comments Within Each Category of the Women's Health Conference Framework on Chronic Debilitating Conditions

Female specific condition	Number of mentions* n *(%)	More common in women and/or morbidity is higher for women condition	Number of mentions* n *(%)
Female specific total	151 (100)	More common in women and/or morbidity is higher total	129 (100)
Endometriosis	22 (15.6)	Mental health	46 (35.7)
Fibroids	17 (22.3)	Autoimmune disease	26 (20.2)
Menstruation	15 (9.9)	HPV	13 (10.1)
Menopause	15 (9.9)	Trauma/PTSD	13 (10.1)
DES	13 (8.6)	Breast cancer	11 (8.5)
Fertility	12 (7.9)	STIs	8 (6.2)
Pelvic floor	10 (6.6)	Lupus	8 (6.2)
PCOS	9 (6)	Osteoporosis	7 (5.4)
Postpartum depression	8 (5.3)	Myalgic Encephalomyelitis/chronic fatigue syndrome	7 (5.4)
Hysterectomy	7 (4.6)	Fibromyalgia	6 (4.7)
Pregnancy loss	5 (3.3)	Sjögren's syndrome	6 (4.7)
Vaginal health	4 (2.6)	Rheumatoid arthritis	5 (3.9)
Abortion research	3 (2)	Migraines	5 (3.9)
Cancer: endometrial	3 (2)	Herpes	4 (3.1)
Vulvodynia	3 (2)	Intimate partner violence	4 (3.1)
Premenstrual syndrome	1 (0.7)	Thyroid disease	2 (1.6)
Vaginal bleeding	1 (0.7)	Eating disorders	1 (0.8)
Vulvar dysplasia	1 (0.7)	TMJ	1 (0.8)

Potentially understudied in women	Number of mentions* n *(%)	High morbidity in women	Number of mentions* n *(%)
Potentially understudied in women	141 (100)	High morbidity in women	62 (100)
Pain, including treatment	20 (14.1)	Heart disease	12 (19.3)
Environmental exposures (incl. climate change)	19 (13.4)	Substance use	12 (19.3)
COVID-19	14 (9.9)	Hypertension	9 (14.5)
Infection	7 (4.9)	Obesity	4 (6.4)
Osteoarthritis	6 (4.2)		
Dementia/alzheimer's	6 (4.2)		
Genetic disorders	3 (2.1)		
Sickle cell	3 (2.1)		
HIV/AIDS	3 (2.1)		
Musculoskeletal disorders	3 (2.1)		
Eye health	3 (2.1)		
Nicotine use	3 (2.1)		
Lipedema	2 (1.4)		
Sepsis	2 (1.4)		
ADHD	1 (0.7)		
FASD	1 (0.7)		

^a^ Comments may have addressed multiple topics, KWs, and conditions so the column totals may exceed 100%.

ADHD, attention-deficit/hyperactivity disorder; FASD, fetal alcohol spectrum disorder; KW, keyword; PTSD, post-traumatic stress disorder; STIs, sexually transmitted infections; TMJ, temporomandibular muscle/joint disorder.

#### Female specific

More than 150 comments addressed female-specific chronic debilitating conditions. Endometriosis, uterine fibroids, menstruation, menopause, diethylstilbestrol exposure, fertility, and pelvic floor issues were the most cited female-specific conditions in the public comments. Bias against funding for research on female-specific conditions was described.

#### More common in women and/or morbidity is higher in women

Of the 129 identified key words on conditions more common in women and/or with higher morbidity for women, 46 concerned mental health as a key priority for women's health, making it the most common condition mentioned within this subcategory. In this category overall, 18 specific conditions were noted, most frequently autoimmune disease, HPV, trauma and post-traumatic stress disorder, breast cancer, and sexually transmitted infections (STIs), including herpes simplex virus.

Commenters described the co-occurrence of mental health conditions, substance use disorders, and violence with other conditions that affect women across the life-course, including during pregnancy and postpartum. Commenters noted significant barriers that many women face when seeking care for mental health and substance use, including expenses often not covered by health insurance.

#### Occur in both sexes, potentially understudied in women

The RFI comments addressed 16 conditions that occur in both sexes but are potentially understudied in women. The most common condition was pain (including chronic pain and pain treatments), followed by environmental exposures (including climate change), COVID-19, infections (including STIs), osteoarthritis, and Alzheimer's disease. Comments described gaps in prevention, screening, diagnosis, and treatment for these conditions. Commenters also noted racial, geographic, and socioeconomic disparities in many conditions within this category, including chronic pain.

#### High morbidity for women

The four conditions with high morbidity for women that were specifically noted included heart disease, substance use, hypertension, and obesity. Comments from the general public, advocacy groups, and researchers addressed health disparities in these conditions and urged additional research on the role of social and structural determinants of health in conditions with high morbidity for women. Public comments about conditions in this category urged advancing holistic, multidimensional approaches and engaging diverse and underrepresented populations of women, in research.

#### Multimorbidity

Many commenters described their experiences living with multimorbidities and their frustrations and challenges with receiving appropriate diagnoses, therapies, and high-quality care. Several members of the biomedical research community and advocacy groups called for increased focus on co- and multimorbidity in women across condition category. These included calls to study the impact and interactions of comorbidities to better understand the mechanisms by which environmental exposures and comorbidities influence disease.

### Cervical cancer

Twenty-seven comments mentioned cervical cancer, focusing on survival rate, treatment, screening, support, and HPV. Of these comments, 24 specifically mentioned treatment, 14 emphasized screening, 13 referenced access to care, and 11 mentioned vaccinations. Specific to cervical cancer survival, concerns were raised regarding persistent racial disparities in cervical cancer survival rates and the causes of these disparities. Comments also highlighted the importance of efforts to increase cervical cancer screening, prevention through HPV vaccination, and attention to social determinants of health and their impact on cancer incidence and mortality in historically excluded and marginalized populations.

### Additional topics

Forty-four percent of comments (*n* = 109) raised issues of relevance to the health of women other than the three topics specifically named in the RFI. Within these comments, 48 keywords were identified. The keywords appearing in this subset of comments included the following: gender inequity (*n* = 6), sex differences (*n* = 5), menstruation (*n* = 5), sex and gender disparities (*n* = 5), SABV (*n* = 4), patient engagement (*n* = 4), diversity in research (*n* = 3), and provider training (*n* = 3).

## Discussion

The NIH continually renews its long-standing commitment to research on the health of women, responds to public health and scientific priorities, and sets out a blueprint for health research priorities. The current framework for advancing the NIH vision for women's health research, as described in *Advancing Science for the Health of Women: 2019–2023 Trans-NIH Strategic Plan for Women's Health Research*, includes achieving a world in which the biomedical research enterprise fully integrates sex and gender influences, every woman receives evidence-based disease prevention and treatment tailored to her own needs, and women in scientific careers reach their full potential.^17^

Despite NIH investments in women's health research, significant gaps were perceived by public stakeholders responding to this RFI. Comments returned to ORWH in response to the RFI (86 FR 35099)^14^ demonstrate a broad range of concerns related to the health of women, including but not limited to MMM, CDCW, and cervical cancer. A wide array of commenters included patients, advocacy groups, and academic and professional organizations originating from geographically diverse locations. The number of comments received for this RFI is more than twice what was received for other recent ORWH RFIs (*e.g.*, NOT-OD-22-186^18^ received 118 responses), indicating high stakeholder interest in identifying research gaps and opportunities related to the health of women.

A significant proportion of comments called for centering research on health equity, referencing increasing rates of MMM, CDCW, and cervical cancer in women of color. Comments calling for increased attention to prenatal, postnatal, and infant care for pregnant Black women and birthing people outlined numerous avenues for future research and align clearly with NIH and ORWH programming, including those research activities focused on understudied, underreported, and underrepresented populations of women and on a life course perspective on the health of women.

Patients who provided comment often described frustrations with health care systems for having ignored or dismissed their symptoms, and many expressed strong desires to contribute to setting NIH research priorities, given that their suffering had gone unrecognized. The comments suggest a potential opportunity for additional engagement with the public to set research priorities for women's health.

Strengths of this analysis include the range of RFI responses received. Diverse interests were well represented in the public comments. Stakeholders submitted comprehensive and detailed comments that reflected the breadth of topics and issues that influence women's health. One limitation of this analysis is its reliance on comments submitted in response to the RFI, as despite extensive promotion of this feedback opportunity, notice of the RFI may not have reached all relevant constituencies. Additional opportunities to gather broad and diverse input could be pursued (*e.g.*, public listening sessions, a community advisory body for the ORWH) to ensure that all interests are heard. An additional limitation relates to the geographic distribution of respondents. Most of the U.S.-based commenters who provided their location were responding from states that have had historically high levels of NIH funding (*e.g.*, California, New York, Maryland). Moving forward, it will be important to outreach to the public and research communities in underrepresented states.

## Implications for Practice and/or Policy

Contemporary public perspectives related to NIH priorities on research on women's health have not been well-described. A diverse group representing multiple public perspectives expressed a broad range of concerns related to women's health, MMM, CDCW, and cervical cancer. The public perception of gaps and opportunities related to women's health can provide valuable guidance to inform public support for research.

## Conclusion

Although 10.8% of the FY2020 NIH budget supports women's health research, public stakeholders perceived ongoing and significant gaps. Good scientific stewardship of the NIH mission of “turning discovery into health” requires broad and diverse input from multiple constituents, including the public, research communities, government agencies, advocacy groups, and professional societies. Diverse perspectives and broad input lead to better science. The comments received in response to this RFI reflect a strong desire from the public for prioritization of NIH research on the health of women. NIH and ORWH look forward to incorporating this feedback in future strategic planning and program development. Formal and informal engagement with stakeholders is critical to attaining the NIH vision of a world where every woman receives evidence-based disease prevention and treatment tailored to her unique needs, desires, and circumstances.
